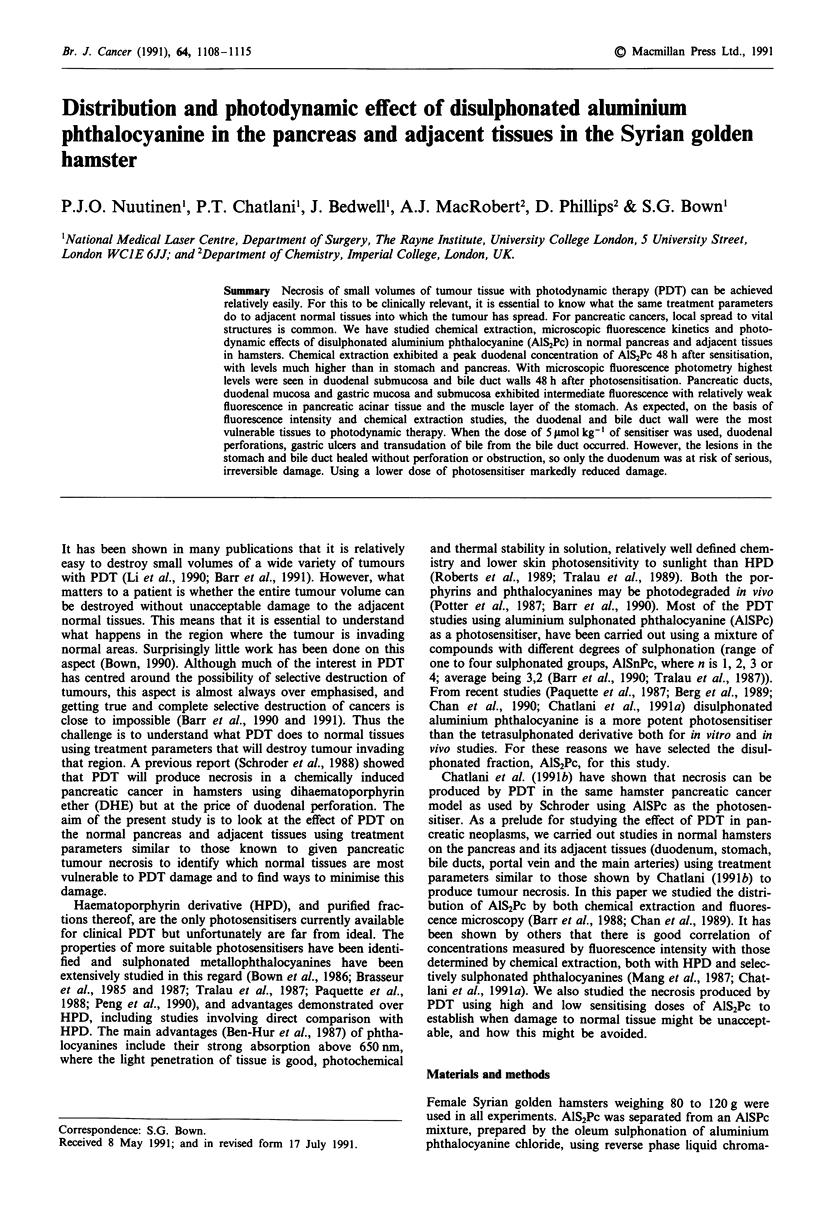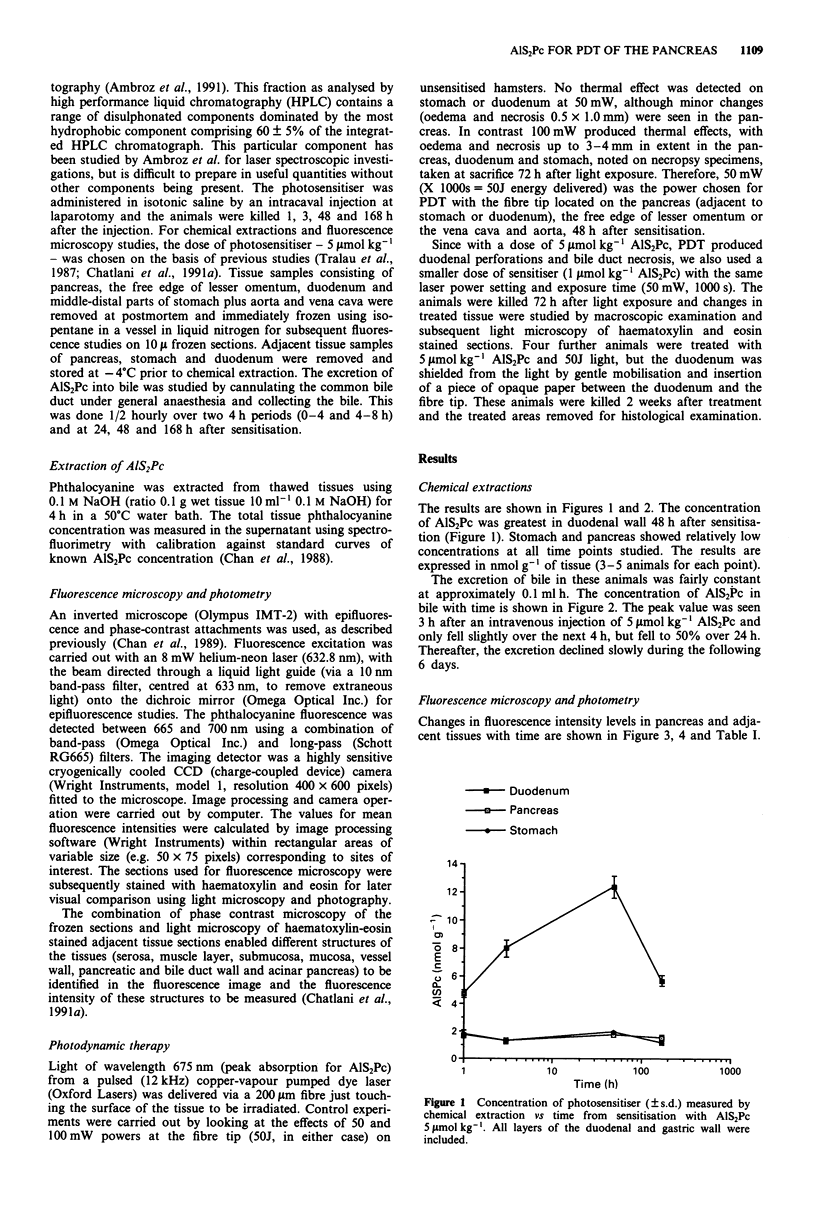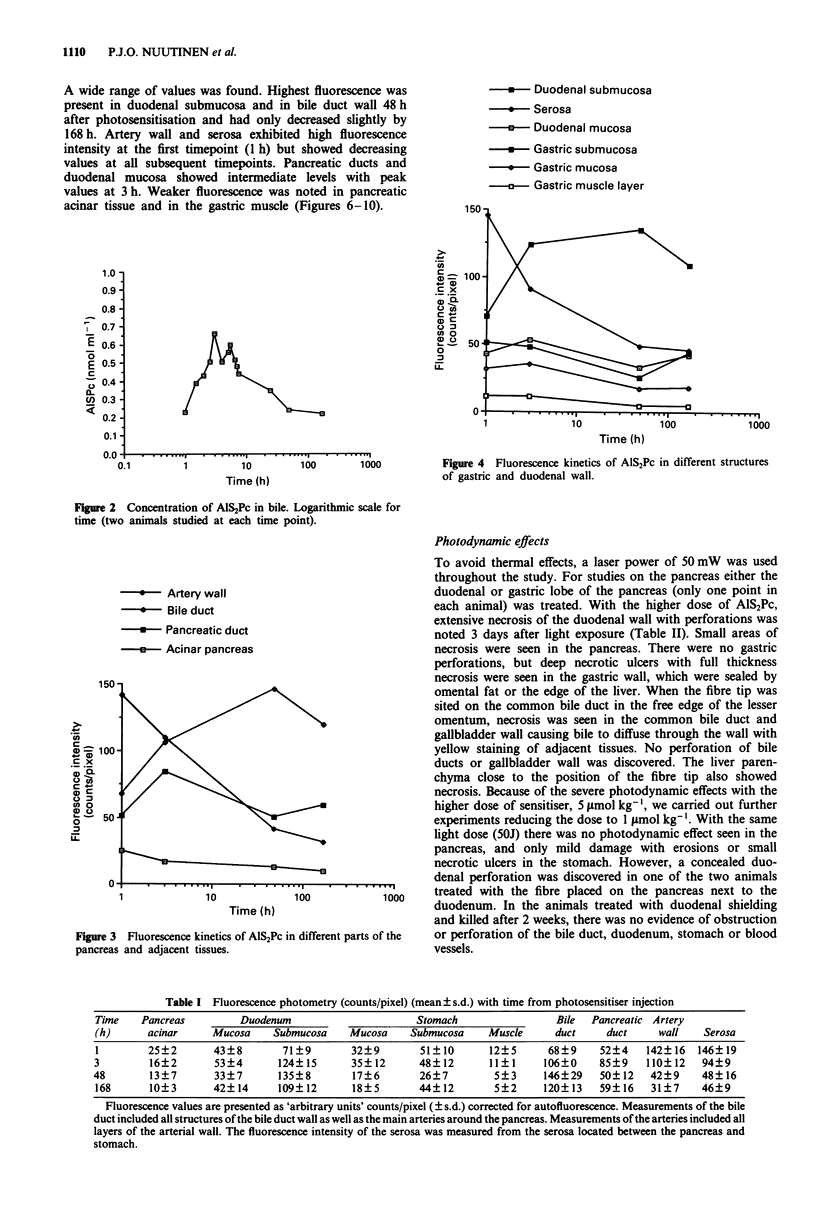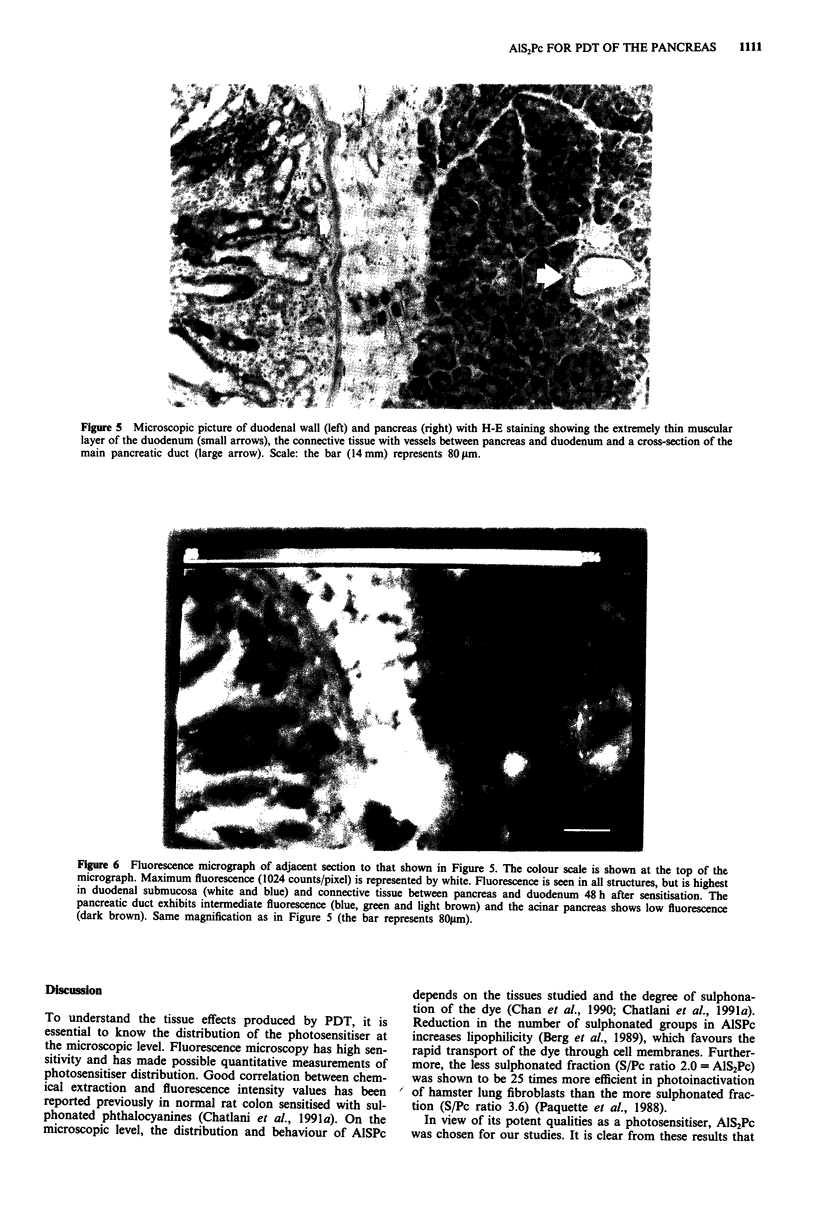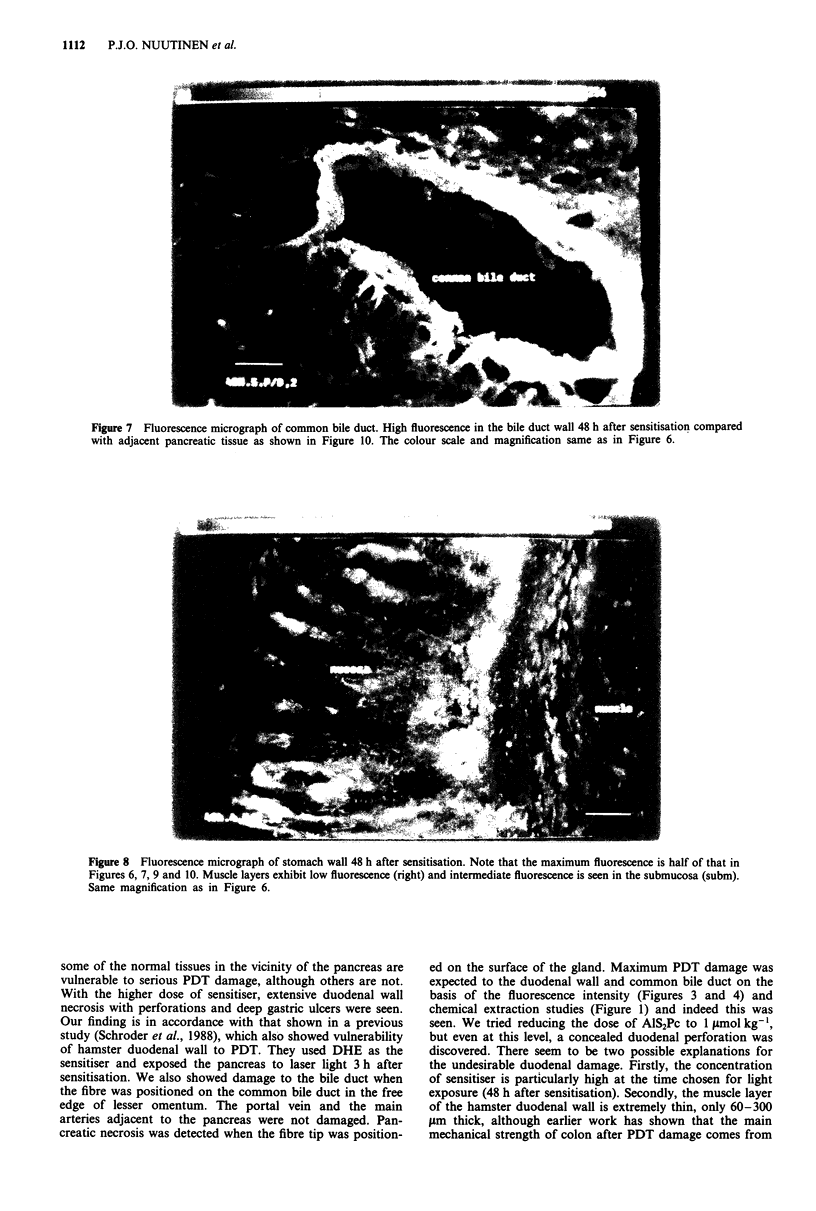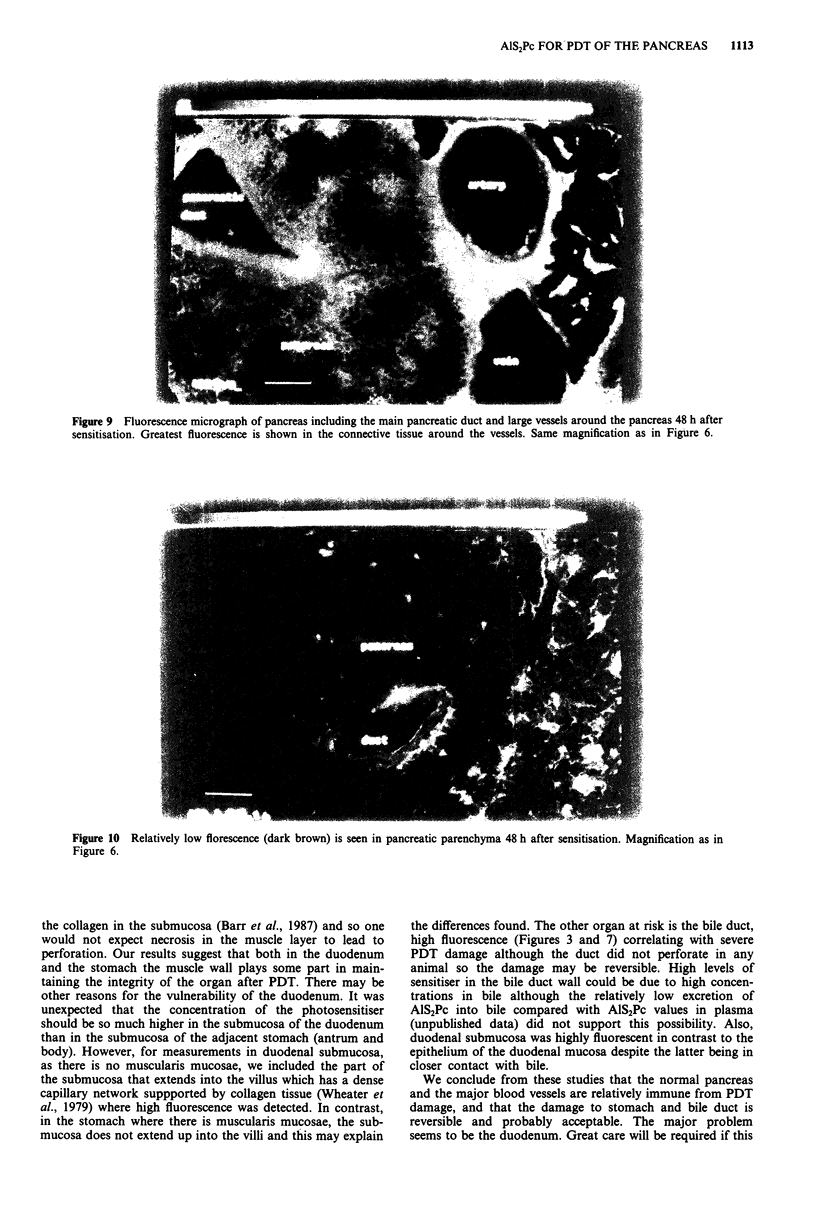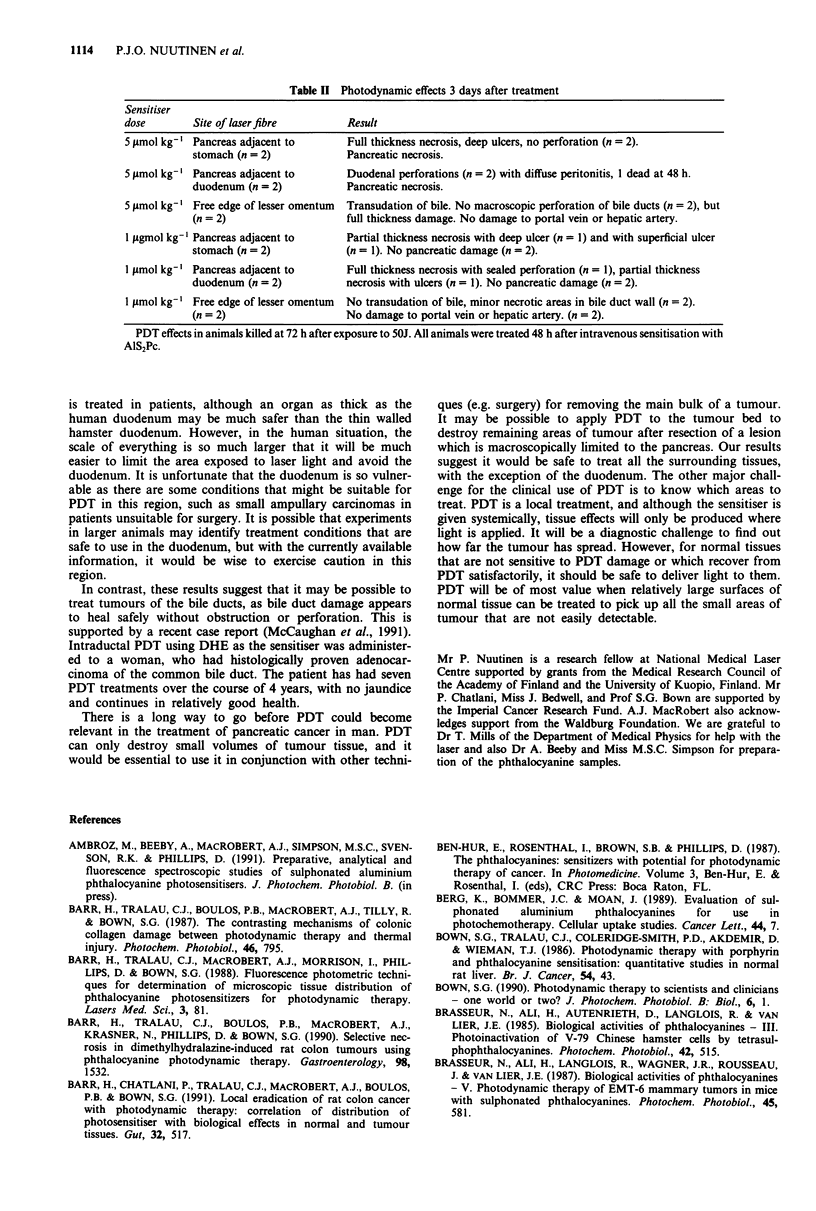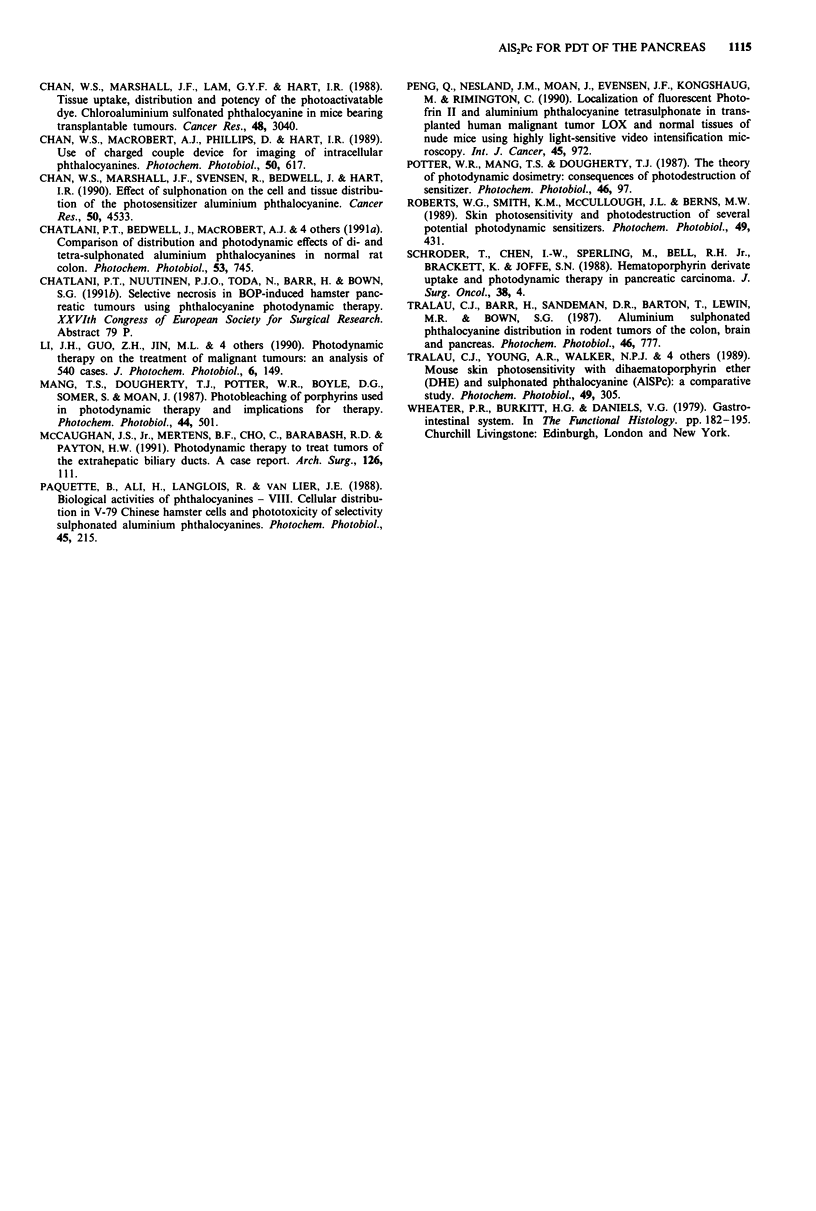# Distribution and photodynamic effect of disulphonated aluminium phthalocyanine in the pancreas and adjacent tissues in the Syrian golden hamster.

**DOI:** 10.1038/bjc.1991.473

**Published:** 1991-12

**Authors:** P. J. Nuutinen, P. T. Chatlani, J. Bedwell, A. J. MacRobert, D. Phillips, S. G. Bown

**Affiliations:** Department of Surgery, Rayne Institute, University College London, UK.

## Abstract

**Images:**


Br. J. Cancer (1991), 64, 1108–1115 © Macmillan Press Ltd., 1991

### Distribution and photodynamic effect of disulphonated aluminium phthalocyanine in the pancreas and adjacent tissues in the Syrian golden hamster

P.J.O. Nuutinen[1], P.T. Chatlani[1], J. Bedwell[1], A.J. MacRobert[2], D. Phillips[2] & S.G. Bown[1]

[1]*National Medical Laser Centre, Department of Surgery, The Rayne Institute, University College London, 5 University Street, London WC1E 6JJ; and* [2]*Department of Chemistry, Imperial College, London, UK.*

**Summary** Necrosis of small volumes of tumour tissue with photodynamic therapy (PDT) can be achieved relatively easily. For this to be clinically relevant, it is essential to know what the same treatment parameters do to adjacent normal tissues into which the tumour has spread. For pancreatic cancers, local spread to vital structures is common. We have studied chemical extraction, microscopic fluorescence kinetics and photodynamic effects of disulphonated aluminium phthalocyanine ($AlS_2Pc$) in normal pancreas and adjacent tissues in hamsters. Chemical extraction exhibited a peak duodenal concentration of $AlS_2Pc$ 48 h after sensitisation, with levels much higher than in stomach and pancreas. With microscopic fluorescence photometry highest levels were seen in duodenal submucosa and bile duct walls 48 h after photosensitisation. Pancreatic ducts, duodenal mucosa and gastric mucosa and submucosa exhibited intermediate fluorescence with relatively weak fluorescence in pancreatic acinar tissue and the muscle layer of the stomach. As expected, on the basis of fluorescence intensity and chemical extraction studies, the duodenal and bile duct wall were the most vulnerable tissues to photodynamic therapy. When the dose of 5 $\mu$mol kg$^{-1}$ of sensitiser was used, duodenal perforations, gastric ulcers and transudation of bile from the bile duct occurred. However, the lesions in the stomach and bile duct healed without perforation or obstruction, so only the duodenum was at risk of serious, irreversible damage. Using a lower dose of photosensitiser markedly reduced damage.

It has been shown in many publications that it is relatively easy to destroy small volumes of a wide variety of tumours with PDT (Li *et al.*, 1990; Barr *et al.*, 1991). However, what matters to a patient is whether the entire tumour volume can be destroyed without unacceptable damage to the adjacent normal tissues. This means that it is essential to understand what happens in the region where the tumour is invading normal areas. Surprisingly little work has been done on this aspect (Bown, 1990). Although much of the interest in PDT has centred around the possibility of selective destruction of tumours, this aspect is almost always over emphasised, and getting true and complete selective destruction of cancers is close to impossible (Barr *et al.*, 1990 and 1991). Thus the challenge is to understand what PDT does to normal tissues using treatment parameters that will destroy tumour invading that region. A previous report (Schroder *et al.*, 1988) showed that PDT will produce necrosis in a chemically induced pancreatic cancer in hamsters using dihaematoporphyrin ether (DHE) but at the price of duodenal perforation. The aim of the present study is to look at the effect of PDT on the normal pancreas and adjacent tissues using treatment parameters similar to those known to given pancreatic tumour necrosis to identify which normal tissues are most vulnerable to PDT damage and to find ways to minimise this damage.

Haematoporphyrin derivative (HPD), and purified fractions thereof, are the only photosensitisers currently available for clinical PDT but unfortunately are far from ideal. The properties of more suitable photosensitisers have been identified and sulphonated metallophthalocyanines have been extensively studied in this regard (Bown *et al.*, 1986; Brasseur *et al.*, 1985 and 1987; Tralau *et al.*, 1987; Paquette *et al.*, 1988; Peng *et al.*, 1990), and advantages demonstrated over HPD, including studies involving direct comparison with HPD. The main advantages (Ben-Hur *et al.*, 1987) of phthalocyanines include their strong absorption above 650 nm, where the light penetration of tissue is good, photochemical and thermal stability in solution, relatively well defined chemistry and lower skin photosensitivity to sunlight than HPD (Roberts *et al.*, 1989; Tralau *et al.*, 1989). Both the porphyrins and phthalocyanines may be photodegraded *in vivo* (Potter *et al.*, 1987; Barr *et al.*, 1990). Most of the PDT studies using aluminium sulphonated phthalocyanine (AlSPc) as a photosensitiser, have been carried out using a mixture of compounds with different degrees of sulphonation (range of one to four sulphonated groups, $AlS_nPc$, where $n$ is 1, 2, 3 or 4; average being 3,2 (Barr *et al.*, 1990; Tralau *et al.*, 1987)). From recent studies (Paquette *et al.*, 1987; Berg *et al.*, 1989; Chan *et al.*, 1990; Chatlani *et al.*, 1991*a*) disulphonated aluminium phthalocyanine is a more potent photosensitiser than the tetrasulphonated derivative both for *in vitro* and *in vivo* studies. For these reasons we have selected the disulphonated fraction, $AlS_2Pc$, for this study.

Chatlani *et al.* (1991*b*) have shown that necrosis can be produced by PDT in the same hamster pancreatic cancer model as used by Schroder using AlSPc as the photosensitiser. As a prelude for studying the effect of PDT in pancreatic neoplasms, we carried out studies in normal hamsters on the pancreas and its adjacent tissues (duodenum, stomach, bile ducts, portal vein and the main arteries) using treatment parameters similar to those shown by Chatlani (1991*b*) to produce tumour necrosis. In this paper we studied the distribution of $AlS_2Pc$ by both chemical extraction and fluorescence microscopy (Barr *et al.*, 1988; Chan *et al.*, 1989). It has been shown by others that there is good correlation of concentrations measured by fluorescence intensity with those determined by chemical extraction, both with HPD and selectively sulphonated phthalocyanines (Mang *et al.*, 1987; Chatlani *et al.*, 1991*a*). We also studied the necrosis produced by PDT using high and low sensitising doses of $AlS_2Pc$ to establish when damage to normal tissue might be unacceptable, and how this might be avoided.

### Materials and methods

Female Syrian golden hamsters weighing 80 to 120 g were used in all experiments. $AlS_2Pc$ was separated from an AlSPc mixture, prepared by the oleum sulphonation of aluminium phthalocyanine chloride, using reverse phase liquid chroma-

Correspondence: S.G. Bown.
Received 8 May 1991; and in revised form 17 July 1991.

tography (Ambroz *et al.*, 1991). This fraction as analysed by high performance liquid chromatography (HPLC) contains a range of disulphonated components dominated by the most hydrophobic component comprising 60 ± 5% of the integrated HPLC chromatograph. This particular component has been studied by Ambroz *et al.* for laser spectroscopic investigations, but is difficult to prepare in useful quantities without other components being present. The photosensitiser was administered in isotonic saline by an intracaval injection at laparotomy and the animals were killed 1, 3, 48 and 168 h after the injection. For chemical extractions and fluorescence microscopy studies, the dose of photosensitiser – 5 $\mu$mol $kg^{-1}$ – was chosen on the basis of previous studies (Tralau *et al.*, 1987; Chatlani *et al.*, 1991*a*). Tissue samples consisting of pancreas, the free edge of lesser omentum, duodenum and middle-distal parts of stomach plus aorta and vena cava were removed at postmortem and immediately frozen using isopentane in a vessel in liquid nitrogen for subsequent fluorescence studies on 10 $\mu$ frozen sections. Adjacent tissue samples of pancreas, stomach and duodenum were removed and stored at –4°C prior to chemical extraction. The excretion of $AlS_2Pc$ into bile was studied by cannulating the common bile duct under general anaesthesia and collecting the bile. This was done 1/2 hourly over two 4 h periods (0–4 and 4–8 h) and at 24, 48 and 168 h after sensitisation.

*Extraction of $AlS_2Pc$*

Phthalocyanine was extracted from thawed tissues using 0.1 M NaOH (ratio 0.1 g wet tissue 10 $ml^{-1}$ 0.1 M NaOH) for 4 h in a 50°C water bath. The total tissue phthalocyanine concentration was measured in the supernatant using spectrofluorimetry with calibration against standard curves of known $AlS_2Pc$ concentration (Chan *et al.*, 1988).

*Fluorescence microscopy and photometry*

An inverted microscope (Olympus IMT-2) with epifluorescence and phase-contrast attachments was used, as described previously (Chan *et al.*, 1989). Fluorescence excitation was carried out with an 8 mW helium-neon laser (632.8 nm), with the beam directed through a liquid light guide (via a 10 nm band-pass filter, centred at 633 nm, to remove extraneous light) onto the dichroic mirror (Omega Optical Inc.) for epifluorescence studies. The phthalocyanine fluorescence was detected between 665 and 700 nm using a combination of band-pass (Omega Optical Inc.) and long-pass (Schott RG665) filters. The imaging detector was a highly sensitive cryogenically cooled CCD (charge-coupled device) camera (Wright Instruments, model 1, resolution 400 × 600 pixels) fitted to the microscope. Image processing and camera operation were carried out by computer. The values for mean fluorescence intensities were calculated by image processing software (Wright Instruments) within rectangular areas of variable size (e.g. 50 × 75 pixels) corresponding to sites of interest. The sections used for fluorescence microscopy were subsequently stained with haematoxylin and eosin for later visual comparison using light microscopy and photography.

The combination of phase contrast microscopy of the frozen sections and light microscopy of haematoxylin-eosin stained adjacent tissue sections enabled different structures of the tissues (serosa, muscle layer, submucosa, mucosa, vessel wall, pancreatic and bile duct wall and acinar pancreas) to be identified in the fluorescence image and the fluorescence intensity of these structures to be measured (Chatlani *et al.*, 1991*a*).

*Photodynamic therapy*

Light of wavelength 675 nm (peak absorption for $AlS_2Pc$) from a pulsed (12 kHz) copper-vapour pumped dye laser (Oxford Lasers) was delivered via a 200 $\mu$m fibre just touching the surface of the tissue to be irradiated. Control experiments were carried out by looking at the effects of 50 and 100 mW powers at the fibre tip (50J, in either case) on unsensitised hamsters. No thermal effect was detected on stomach or duodenum at 50 mW, although minor changes (oedema and necrosis 0.5 × 1.0 mm) were seen in the pancreas. In contrast 100 mW produced thermal effects, with oedema and necrosis up to 3–4 mm in extent in the pancreas, duodenum and stomach, noted on necropsy specimens, taken at sacrifice 72 h after light exposure. Therefore, 50 mW (X 1000s = 50J energy delivered) was the power chosen for PDT with the fibre tip located on the pancreas (adjacent to stomach or duodenum), the free edge of lesser omentum or the vena cava and aorta, 48 h after sensitisation.

Since with a dose of 5 $\mu$mol $kg^{-1}$ $AlS_2Pc$, PDT produced duodenal perforations and bile duct necrosis, we also used a smaller dose of sensitiser (1 $\mu$mol $kg^{-1}$ $AlS_2Pc$) with the same laser power setting and exposure time (50 mW, 1000 s). The animals were killed 72 h after light exposure and changes in treated tissue were studied by macroscopic examination and subsequent light microscopy of haematoxylin and eosin stained sections. Four further animals were treated with 5 $\mu$mol $kg^{-1}$ $AlS_2Pc$ and 50J light, but the duodenum was shielded from the light by gentle mobilisation and insertion of a piece of opaque paper between the duodenum and the fibre tip. These animals were killed 2 weeks after treatment and the treated areas removed for histological examination.

### Results

*Chemical extractions*

The results are shown in Figures 1 and 2. The concentration of $AlS_2Pc$ was greatest in duodenal wall 48 h after sensitisation (Figure 1). Stomach and pancreas showed relatively low concentrations at all time points studied. The results are expressed in nmol $g^{-1}$ of tissue (3–5 animals for each point).

The excretion of bile in these animals was fairly constant at approximately 0.1 ml h. The concentration of $AlS_2Pc$ in bile with time is shown in Figure 2. The peak value was seen 3 h after an intravenous injection of 5 $\mu$mol $kg^{-1}$ $AlS_2Pc$ and only fell slightly over the next 4 h, but fell to 50% over 24 h. Thereafter, the excretion declined slowly during the following 6 days.

*Fluorescence microscopy and photometry*

Changes in fluorescence intensity levels in pancreas and adjacent tissues with time are shown in Figure 3, 4 and Table I.

**Figure 1** Concentration of photosensitiser (±s.d.) measured by chemical extraction *vs* time from sensitisation with $AlS_2Pc$ 5 $\mu$mol $kg^{-1}$. All layers of the duodenal and gastric wall were included.

A wide range of values was found. Highest fluorescence was present in duodenal submucosa and in bile duct wall 48 h after photosensitisation and had only decreased slightly by 168 h. Artery wall and serosa exhibited high fluorescence intensity at the first timepoint (1 h) but showed decreasing values at all subsequent timepoints. Pancreatic ducts and duodenal mucosa showed intermediate levels with peak values at 3 h. Weaker fluorescence was noted in pancreatic acinar tissue and in the gastric muscle (Figures 6–10).

**Figure 2** Concentration of $AlS_2Pc$ in bile. Logarithmic scale for time (two animals studied at each time point).

**Figure 3** Fluorescence kinetics of $AlS_2Pc$ in different parts of the pancreas and adjacent tissues.

**Figure 4** Fluorescence kinetics of $AlS_2Pc$ in different structures of gastric and duodenal wall.

*Photodynamic effects*

To avoid thermal effects, a laser power of 50 mW was used throughout the study. For studies on the pancreas either the duodenal or gastric lobe of the pancreas (only one point in each animal) was treated. With the higher dose of $AlS_2Pc$, extensive necrosis of the duodenal wall with perforations was noted 3 days after light exposure (Table II). Small areas of necrosis were seen in the pancreas. There were no gastric perforations, but deep necrotic ulcers with full thickness necrosis were seen in the gastric wall, which were sealed by omental fat or the edge of the liver. When the fibre tip was sited on the common bile duct in the free edge of the lesser omentum, necrosis was seen in the common bile duct and gallbladder wall causing bile to diffuse through the wall with yellow staining of adjacent tissues. No perforation of bile ducts or gallbladder wall was discovered. The liver parenchyma close to the position of the fibre tip also showed necrosis. Because of the severe photodynamic effects with the higher dose of sensitiser, 5 $\mu$mol $kg^{-1}$, we carried out further experiments reducing the dose to 1 $\mu$mol $kg^{-1}$. With the same light dose (50J) there was no photodynamic effect seen in the pancreas, and only mild damage with erosions or small necrotic ulcers in the stomach. However, a concealed duodenal perforation was discovered in one of the two animals treated with the fibre placed on the pancreas next to the duodenum. In the animals treated with duodenal shielding and killed after 2 weeks, there was no evidence of obstruction or perforation of the bile duct, duodenum, stomach or blood vessels.

**Table I** Fluorescence photometry (counts/pixel) (mean ± s.d.) with time from photosensitiser injection

| *Time* | *Pancreas* | *Duodenum* | | *Stomach* | | | *Bile* | *Pancreatic* | *Artery* | |
|---|---|---|---|---|---|---|---|---|---|---|
| *(h)* | *acinar* | *Mucosa* | *Submucosa* | *Mucosa* | *Submucosa* | *Muscle* | *duct* | *duct* | *wall* | *Serosa* |
| 1 | 25±2 | 43±8 | 71±9 | 32±9 | 51±10 | 12±5 | 68±9 | 52±4 | 142±16 | 146±19 |
| 3 | 16±2 | 53±4 | 124±15 | 35±12 | 48±12 | 11±1 | 106±0 | 85±9 | 110±12 | 94±9 |
| 48 | 13±7 | 33±7 | 135±8 | 17±6 | 26±7 | 5±3 | 146±29 | 50±12 | 42±9 | 48±16 |
| 168 | 10±3 | 42±14 | 109±12 | 18±5 | 44±12 | 5±2 | 120±13 | 59±16 | 31±7 | 46±9 |

Fluorescence values are presented as 'arbitrary units' counts/pixel (±s.d.) corrected for autofluorescence. Measurements of the bile duct included all structures of the bile duct wall as well as the main arteries around the pancreas. Measurements of the arteries included all layers of the arterial wall. The fluorescence intensity of the serosa was measured from the serosa located between the pancreas and stomach.

**Figure 5** Microscopic picture of duodenal wall (left) and pancreas (right) with H-E staining showing the extremely thin muscular layer of the duodenum (small arrows), the connective tissue with vessels between pancreas and duodenum and a cross-section of the main pancreatic duct (large arrow). Scale: the bar (14 mm) represents 80 μm.

**Figure 6** Fluorescence micrograph of adjacent section to that shown in Figure 5. The colour scale is shown at the top of the micrograph. Maximum fluorescence (1024 counts/pixel) is represented by white. Fluorescence is seen in all structures, but is highest in duodenal submucosa (white and blue) and connective tissue between pancreas and duodenum 48 h after sensitisation. The pancreatic duct exhibits intermediate fluorescence (blue, green and light brown) and the acinar pancreas shows low fluorescence (dark brown). Same magnification as in Figure 5 (the bar represents 80μm).

### Discussion

To understand the tissue effects produced by PDT, it is essential to know the distribution of the photosensitiser at the microscopic level. Fluorescence microscopy has high sensitivity and has made possible quantitative measurements of photosensitiser distribution. Good correlation between chemical extraction and fluorescence intensity values has been reported previously in normal rat colon sensitised with sulphonated phthalocyanines (Chatlani *et al.*, 1991*a*). On the microscopic level, the distribution and behaviour of AlSPc depends on the tissues studied and the degree of sulphonation of the dye (Chan *et al.*, 1990; Chatlani *et al.*, 1991*a*). Reduction in the number of sulphonated groups in AlSPc increases lipophilicity (Berg *et al.*, 1989), which favours the rapid transport of the dye through cell membranes. Furthermore, the less sulphonated fraction (S/Pc ratio 2.0 = $AlS_2Pc$) was shown to be 25 times more efficient in photoinactivation of hamster lung fibroblasts than the more sulphonated fraction (S/Pc ratio 3.6) (Paquette *et al.*, 1988).

In view of its potent qualities as a photosensitiser, $AlS_2Pc$ was chosen for our studies. It is clear from these results that

**Figure 7** Fluorescence micrograph of common bile duct. High fluorescence in the bile duct wall 48 h after sensitisation compared with adjacent pancreatic tissue as shown in Figure 10. The colour scale and magnification same as in Figure 6.

**Figure 8** Fluorescence micrograph of stomach wall 48 h after sensitisation. Note that the maximum fluorescence is half of that in Figures 6, 7, 9 and 10. Muscle layers exhibit low fluorescence (right) and intermediate fluorescence is seen in the submucosa (subm). Same magnification as in Figure 6.

some of the normal tissues in the vicinity of the pancreas are vulnerable to serious PDT damage, although others are not. With the higher dose of sensitiser, extensive duodenal wall necrosis with perforations and deep gastric ulcers were seen. Our finding is in accordance with that shown in a previous study (Schroder *et al.*, 1988), which also showed vulnerability of hamster duodenal wall to PDT. They used DHE as the sensitiser and exposed the pancreas to laser light 3 h after sensitisation. We also showed damage to the bile duct when the fibre was positioned on the common bile duct in the free edge of lesser omentum. The portal vein and the main arteries adjacent to the pancreas were not damaged. Pancreatic necrosis was detected when the fibre tip was positioned on the surface of the gland. Maximum PDT damage was expected to the duodenal wall and common bile duct on the basis of the fluorescence intensity (Figures 3 and 4) and chemical extraction studies (Figure 1) and indeed this was seen. We tried reducing the dose of $AlS_2Pc$ to 1 $\mu mol\ kg^{-1}$, but even at this level, a concealed duodenal perforation was discovered. There seem to be two possible explanations for the undesirable duodenal damage. Firstly, the concentration of sensitiser is particularly high at the time chosen for light exposure (48 h after sensitisation). Secondly, the muscle layer of the hamster duodenal wall is extremely thin, only 60–300 $\mu m$ thick, although earlier work has shown that the main mechanical strength of colon after PDT damage comes from

**Figure 9** Fluorescence micrograph of pancreas including the main pancreatic duct and large vessels around the pancreas 48 h after sensitisation. Greatest fluorescence is shown in the connective tissue around the vessels. Same magnification as in Figure 6.

**Figure 10** Relatively low florescence (dark brown) is seen in pancreatic parenchyma 48 h after sensitisation. Magnification as in Figure 6.

the collagen in the submucosa (Barr *et al.*, 1987) and so one would not expect necrosis in the muscle layer to lead to perforation. Our results suggest that both in the duodenum and the stomach the muscle wall plays some part in maintaining the integrity of the organ after PDT. There may be other reasons for the vulnerability of the duodenum. It was unexpected that the concentration of the photosensitiser should be so much higher in the submucosa of the duodenum than in the submucosa of the adjacent stomach (antrum and body). However, for measurements in duodenal submucosa, as there is no muscularis mucosae, we included the part of the submucosa that extends into the villus which has a dense capillary network suppported by collagen tissue (Wheater *et al.*, 1979) where high fluorescence was detected. In contrast, in the stomach where there is muscularis mucosae, the submucosa does not extend up into the villi and this may explain the differences found. The other organ at risk is the bile duct, high fluorescence (Figures 3 and 7) correlating with severe PDT damage although the duct did not perforate in any animal so the damage may be reversible. High levels of sensitiser in the bile duct wall could be due to high concentrations in bile although the relatively low excretion of $AlS_2Pc$ into bile compared with $AlS_2Pc$ values in plasma (unpublished data) did not support this possibility. Also, duodenal submucosa was highly fluorescent in contrast to the epithelium of the duodenal mucosa despite the latter being in closer contact with bile.

We conclude from these studies that the normal pancreas and the major blood vessels are relatively immune from PDT damage, and that the damage to stomach and bile duct is reversible and probably acceptable. The major problem seems to be the duodenum. Great care will be required if this

**Table II** Photodynamic effects 3 days after treatment

| *Sensitiser dose* | *Site of laser fibre* | *Result* |
|---|---|---|
| 5 μmol $kg^{-1}$ | Pancreas adjacent to stomach ($n = 2$) | Full thickness necrosis, deep ulcers, no perforation ($n = 2$). Pancreatic necrosis. |
| 5 μmol $kg^{-1}$ | Pancreas adjacent to duodenum ($n = 2$) | Duodenal perforations ($n = 2$) with diffuse peritonitis, 1 dead at 48 h. Pancreatic necrosis. |
| 5 μmol $kg^{-1}$ | Free edge of lesser omentum ($n = 2$) | Transudation of bile. No macroscopic perforation of bile ducts ($n = 2$), but full thickness damage. No damage to portal vein or hepatic artery. |
| 1 μgmol $kg^{-1}$ | Pancreas adjacent to stomach ($n = 2$) | Partial thickness necrosis with deep ulcer ($n = 1$) and with superficial ulcer ($n = 1$). No pancreatic damage ($n = 2$). |
| 1 μmol $kg^{-1}$ | Pancreas adjacent to duodenum ($n = 2$) | Full thickness necrosis with sealed perforation ($n = 1$), partial thickness necrosis with ulcers ($n = 1$). No pancreatic damage ($n = 2$). |
| 1 μmol $kg^{-1}$ | Free edge of lesser omentum ($n = 2$) | No transudation of bile, minor necrotic areas in bile duct wall ($n = 2$). No damage to portal vein or hepatic artery. ($n = 2$). |

PDT effects in animals killed at 72 h after exposure to 50J. All animals were treated 48 h after intravenous sensitisation with $AlS_2Pc$.

is treated in patients, although an organ as thick as the human duodenum may be much safer than the thin walled hamster duodenum. However, in the human situation, the scale of everything is so much larger that it will be much easier to limit the area exposed to laser light and avoid the duodenum. It is unfortunate that the duodenum is so vulnerable as there are some conditions that might be suitable for PDT in this region, such as small ampullary carcinomas in patients unsuitable for surgery. It is possible that experiments in larger animals may identify treatment conditions that are safe to use in the duodenum, but with the currently available information, it would be wise to exercise caution in this region.

In contrast, these results suggest that it may be possible to treat tumours of the bile ducts, as bile duct damage appears to heal safely without obstruction or perforation. This is supported by a recent case report (McCaughan *et al.*, 1991). Intraductal PDT using DHE as the sensitiser was administered to a woman, who had histologically proven adenocarcinoma of the common bile duct. The patient has had seven PDT treatments over the course of 4 years, with no jaundice and continues in relatively good health.

There is a long way to go before PDT could become relevant in the treatment of pancreatic cancer in man. PDT can only destroy small volumes of tumour tissue, and it would be essential to use it in conjunction with other techniques (e.g. surgery) for removing the main bulk of a tumour. It may be possible to apply PDT to the tumour bed to destroy remaining areas of tumour after resection of a lesion which is macroscopically limited to the pancreas. Our results suggest it would be safe to treat all the surrounding tissues, with the exception of the duodenum. The other major challenge for the clinical use of PDT is to know which areas to treat. PDT is a local treatment, and although the sensitiser is given systemically, tissue effects will only be produced where light is applied. It will be a diagnostic challenge to find out how far the tumour has spread. However, for normal tissues that are not sensitive to PDT damage or which recover from PDT satisfactorily, it should be safe to deliver light to them. PDT will be of most value when relatively large surfaces of normal tissue can be treated to pick up all the small areas of tumour that are not easily detectable.

Mr P. Nuutinen is a research fellow at National Medical Laser Centre supported by grants from the Medical Research Council of the Academy of Finland and the University of Kuopio, Finland. Mr P. Chatlani, Miss J. Bedwell, and Prof S.G. Bown are supported by the Imperial Cancer Research Fund. A.J. MacRobert also acknowledges support from the Waldburg Foundation. We are grateful to Dr T. Mills of the Department of Medical Physics for help with the laser and also Dr A. Beeby and Miss M.S.C. Simpson for preparation of the phthalocyanine samples.

### References

AMBROZ, M., BEEBY, A., MACROBERT, A.J., SIMPSON, M.S.C., SVENSON, R.K. & PHILLIPS, D. (1991). Preparative, analytical and fluorescence spectroscopic studies of sulphonated aluminium phthalocyanine photosensitisers. *J. Photochem. Photobiol. B.* (in press).

BARR, H., TRALAU, C.J., BOULOS, P.B., MACROBERT, A.J., TILLY, R. & BOWN, S.G. (1987). The contrasting mechanisms of colonic collagen damage between photodynamic therapy and thermal injury. *Photochem. Photobiol.*, **46**, 795.

BARR, H., TRALAU, C.J., MACROBERT, A.J., MORRISON, I., PHILLIPS, D. & BOWN, S.G. (1988). Fluorescence photometric techniques for determination of microscopic tissue distribution of phthalocyanine photosensitizers for photodynamic therapy. *Lasers Med. Sci.*, **3**, 81.

BARR, H., TRALAU, C.J., BOULOS, P.B., MACROBERT, A.J., KRASNER, N., PHILLIPS, D. & BOWN, S.G. (1990). Selective necrosis in dimethylhydralazine-induced rat colon tumours using phthalocyanine photodynamic therapy. *Gastroenterology*, **98**, 1532.

BARR, H., CHATLANI, P., TRALAU, C.J., MACROBERT, A.J., BOULOS, P.B. & BOWN, S.G. (1991). Local eradication of rat colon cancer with photodynamic therapy: correlation of distribution of photosensitiser with biological effects in normal and tumour tissues. *Gut*, **32**, 517.

BEN-HUR, E., ROSENTHAL, I., BROWN, S.B. & PHILLIPS, D. (1987). The phthalocyanines: sensitizers with potential for photodynamic therapy of cancer. In *Photomedicine*. Volume 3, Ben-Hur, E. & Rosenthal, I. (eds), CRC Press: Boca Raton, FL.

BERG, K., BOMMER, J.C. & MOAN, J. (1989). Evaluation of sulphonated aluminium phthalocyanines for use in photochemotherapy. Cellular uptake studies. *Cancer Lett.*, **44**, 7.

BOWN, S.G., TRALAU, C.J., COLERIDGE-SMITH, P.D., AKDEMIR, D. & WIEMAN, T.J. (1986). Photodynamic therapy with porphyrin and phthalocyanine sensitisation: quantitative studies in normal rat liver. *Br. J. Cancer*, **54**, 43.

BOWN, S.G. (1990). Photodynamic therapy to scientists and clinicians – one world or two? *J. Photochem. Photobiol. B: Biol.*, **6**, 1.

BRASSEUR, N., ALI, H., AUTENRIETH, D., LANGLOIS, R. & VAN LIER, J.E. (1985). Biological activities of phthalocyanines – III. Photoinactivation of V-79 Chinese hamster cells by tetrasulphophthalocyanines. *Photochem. Photobiol.*, **42**, 515.

BRASSEUR, N., ALI, H., LANGLOIS, R., WAGNER, J.R., ROUSSEAU, J. & VAN LIER, J.E. (1987). Biological activities of phthalocyanines – V. Photodynamic therapy of EMT-6 mammary tumors in mice with sulphonated phthalocyanines. *Photochem. Photobiol.*, **45**, 581.

CHAN, W.S., MARSHALL, J.F., LAM, G.Y.F. & HART, I.R. (1988). Tissue uptake, distribution and potency of the photoactivatable dye. Chloroaluminium sulfonated phthalocyanine in mice bearing transplantable tumours. *Cancer Res.*, **48**, 3040.

CHAN, W.S., MACROBERT, A.J., PHILLIPS, D. & HART, I.R. (1989). Use of charged couple device for imaging of intracellular phthalocyanines. *Photochem. Photobiol.*, **50**, 617.

CHAN, W.S., MARSHALL, J.F., SVENSEN, R., BEDWELL, J. & HART, I.R. (1990). Effect of sulphonation on the cell and tissue distribution of the photosensitizer aluminium phthalocyanine. *Cancer Res.*, **50**, 4533.

CHATLANI, P.T., BEDWELL, J., MACROBERT, A.J. & 4 others (1991*a*). Comparison of distribution and photodynamic effects of di- and tetra-sulphonated aluminium phthalocyanines in normal rat colon. *Photochem. Photobiol.*, **53**, 745.

CHATLANI, P.T., NUUTINEN, P.J.O., TODA, N., BARR, H. & BOWN, S.G. (1991*b*). Selective necrosis in BOP-induced hamster pancreatic tumours using phthalocyanine photodynamic therapy. *XXVIth Congress of European Society for Surgical Research.* Abstract 79 P.

LI, J.H., GUO, Z.H., JIN, M.L. & 4 others (1990). Photodynamic therapy on the treatment of malignant tumours: an analysis of 540 cases. *J. Photochem. Photobiol.*, **6**, 149.

MANG, T.S., DOUGHERTY, T.J., POTTER, W.R., BOYLE, D.G., SOMER, S. & MOAN, J. (1987). Photobleaching of porphyrins used in photodynamic therapy and implications for therapy. *Photochem. Photobiol.*, **44**, 501.

MCCAUGHAN, J.S., Jr., MERTENS, B.F., CHO, C., BARABASH, R.D. & PAYTON, H.W. (1991). Photodynamic therapy to treat tumors of the extrahepatic biliary ducts. A case report. *Arch. Surg.*, **126**, 111.

PAQUETTE, B., ALI, H., LANGLOIS, R. & VAN LIER, J.E. (1988). Biological activities of phthalocyanines – VIII. Cellular distribution in V-79 Chinese hamster cells and phototoxicity of selectivity sulphonated aluminium phthalocyanines. *Photochem. Photobiol.*, **45**, 215.

PENG, Q., NESLAND, J.M., MOAN, J., EVENSEN, J.F., KONGSHAUG, M. & RIMINGTON, C. (1990). Localization of fluorescent Photofrin II and aluminium phthalocyanine tetrasulphonate in transplanted human malignant tumor LOX and normal tissues of nude mice using highly light-sensitive video intensification microscopy. *Int. J. Cancer*, **45**, 972.

POTTER, W.R., MANG, T.S. & DOUGHERTY, T.J. (1987). The theory of photodynamic dosimetry: consequences of photodestruction of sensitizer. *Photochem. Photobiol.*, **46**, 97.

ROBERTS, W.G., SMITH, K.M., MCCULLOUGH, J.L. & BERNS, M.W. (1989). Skin photosensitivity and photodestruction of several potential photodynamic sensitizers. *Photochem. Photobiol.*, **49**, 431.

SCHRODER, T., CHEN, I.-W., SPERLING, M., BELL, R.H. Jr., BRACKETT, K. & JOFFE, S.N. (1988). Hematoporphyrin derivate uptake and photodynamic therapy in pancreatic carcinoma. *J. Surg. Oncol.*, **38**, 4.

TRALAU, C.J., BARR, H., SANDEMAN, D.R., BARTON, T., LEWIN, M.R. & BOWN, S.G. (1987). Aluminium sulphonated phthalocyanine distribution in rodent tumors of the colon, brain and pancreas. *Photochem. Photobiol.*, **46**, 777.

TRALAU, C.J., YOUNG, A.R., WALKER, N.P.J. & 4 others (1989). Mouse skin photosensitivity with dihaematoporphyrin ether (DHE) and sulphonated phthalocyanine (AlSPc): a comparative study. *Photochem. Photobiol.*, **49**, 305.

WHEATER, P.R., BURKITT, H.G. & DANIELS, V.G. (1979). Gastrointestinal system. In *The Functional Histology*. pp. 182–195. Churchill Livingstone: Edinburgh, London and New York.